# COVID-19 induced liver function abnormality associates with age

**DOI:** 10.18632/aging.103720

**Published:** 2020-07-28

**Authors:** Shasha Li, Jinsong Li, Zhenhua Zhang, Lin Tan, Tuo Shao, Ming Li, Xiuyong Li, Jacinta A. Holmes, Wenyu Lin, Mingfeng Han

**Affiliations:** 1 Department of Hepatology, The Second People’s Hospital of Fuyang, Fuyang 236015, Anhui Province, P.R. China; 2 Department of Hepatology, The Second Hospital of Anhui Medical University, Hefei 236015, Anhui Province, P.R. China; 3 Liver Center and Gastrointestinal Division, Department of Medicine, Massachusetts General Hospital, Harvard Medical School, Boston, MA 02114, USA; 4 Blood Purification Center, The Second People’s Hospital of Fuyang, Fuyang 236015, Anhui Province, P.R. China; 5 Department of Gastroenterology, St Vincent’s Hospital, University of Melbourne, Fitzroy 3065, VIC, Australia; 6 Department of Respiratory, The Second People’s Hospital of Fuyang, Fuyang 236015, Anhui Province, P.R. China

**Keywords:** COVID-19, age, liver abnormality, severe, critical patient

## Abstract

Background: Coronavirus disease 2019 (COVID-19) is a novel infectious disease that may cause fever, dry cough, fatigue and shortness of breath. The impact of COVID-19 on liver function is not well described.

Results: We found that the overall frequency of LFT abnormality was 17.6%. Frequency of LFT abnormality was significantly greater in patients with severe/critical (SC) COVID-19 compared to those with mild/moderate (MM) COVID-19 (32.4% vs 11.6%, p=0.011). Among patients with LFT abnormality, the median age was significantly higher in the SC group compared to the MM group (52 vs 39 years, p=0.021).

Conclusion: COVID-19 is frequently associated with mild liver function abnormality, particularly in individuals with severe/critical COVID-19 who were older. Liver function should be monitored carefully during infection, with judicious use of hepatotoxic agents where possible and avoidance of prolonged hypotension to minimize liver injury in older patients.

Methods: The No. 2 People’s Hospital of Fuyang City in China has admitted a total of 159 patients with confirmed COVID-19 since the outbreak from January 2020 to March 2020. We analyzed the incidence of liver function test (LFT) abnormality in these patients with confirmed COVID-19 infection.

## INTRODUCTION

Coronavirus disease 2019 (COVID-19) is a novel infectious disease caused by the severe acute respiratory syndrome coronavirus 2 (SARS coronavirus 2 or SARS-CoV-2) [1–5]. The COVID-19 outbreak was first described in November/December 2019 in China, and has since spread to over 180 countries around the world [6–10]. Due to this rapid spread and severity of the illness, the World Health Organization characterized COVID-19 as a pandemic [11–13]. COVID-19 continues to be a serious threat to public health worldwide, with a global morality rate of 5.15% as of June 24^th^ 2020 [10]. However, the mortality rate has varied significantly across regions, ranging from low rates in Qatar (0.11%), Russia (1.40%) and South Africa (1.98%), to intermediate rates in India (3.17%), Germany (4.63%), Iran (4.70%), China (5.48%) and the United States (5.16%), to very high rates in Spain (11.48%), the United Kingdom (13.98%), Italy (14.52%), France (15.03%) and Belgium (15.97%) (see the Coronavirus Resource Centre or the latest worldwide data [10, 14]). COVID-19 most commonly causes fever, cough, shortness of breath, myalgia, fatigue, and sore throat [1], ranging from mild in severity to severe, with around a quarter requiring intensive care admission in the largest case series to date [1, 15]. Asymptomatic infection with confirmed transmission and atypical presentations with abdominal pain, nausea, vomiting and diarrhea have also been reported [15]. However, the frequency of liver dysfunction in COVID-19 infection has not been well described, and in particular have been difficult to interpret due to co-administration of hepatotoxic agents and varied timing of liver function abnormality in the course of the illness and across age groups [16–18]. In this brief report, we sought to analyze the association between COVID-19 infection, liver function test (LFT) abnormality and age in the 159 patients hospitalized for confirmed COVID-19 at the No. 2 People’s Hospital of Fuyang City, Fuyang, Anhui Province, China.

## RESULTS

### Patients

A total of 159 patients were admitted with confirmed COVID-19 and enrolled in this study. Baseline demographics and patient characteristics according to severity of COVID-19 are presented in Table 1. Overall, the median age was 43 years, and 56.6% (90/159) were male (Table 1). Thirty-four patients (21.4%) were classified to have severe or critical illness (SC) and the remaining 125 patients (78.6%) were classified to have mild/moderate illness (MM) (Tables 1, 2). In brief, patients in the MM group were significantly younger, had a lower body mass index (BMI), were less likely to have fever, and had a lower heart rate, lower respiratory rate and higher oxygen saturations at admission compared to the SC group (Table 1), reflecting the severity of their COVID-19. There was a significantly higher proportion of patients with underlying chronic hepatitis B virus (HBV) infection in the SC group compared to the MM group (Table 1). There was a significantly higher proportion of patients with hypertension in the SC group compared to the MM group (Table 1). Other comorbidities were similar between both groups. Patients with chronic HBV were treated with entecavir (ETV) if they met the APASL guidelines for HBV treatment [23].

**Table 1:** Comparison of baseline demographics and clinical characteristics between SC and MM groups. Values are expressed as median (interquartile range (IQR), 25-75%). P value is the comparison between severe/critical (SC) and mild/moderate (MM) patients. *P<0.05, **P<0.01, ***P<0.001.

**Characteristic**	**SC**	**MM**	**P value**
**Total number (n, %)**	34 (21.4%)	125 (78.6%)	
**with liver function test abnormality (n, %)**	11 (32.4%)	17 (11.6%)	0.011
**Age (years) (median, IQR)**	49.5 (42.5-65.3)	41.0 (29.0-50.0)	<0.0001
**Male gender (n, %)**	23 (67.6%)	67 (53.6%)	0.143
**Fever (n, %)**	34(100%)	84(67.2%)	<0.0001
**Temperature (°C) (Median, IQR)**	37.1 (36.8-37.9)	36.8 (36.5-37.5)	0.014
**Heart rate** **(beats / minute) (Median, IQR)**	96 (78-102)	84 (80-91)	0.039
**Blood Pressure (mmHg) (Median, IQR)**	130 (116-142)/84 (73-93)	128 (119.5-140)/85 (75.5-92)	0.671/0.711
**Respiratory rate (breaths / minute) (Median, IQR)**	20 (19-23)	20 (19-21.5)	0.031
**Oxygen saturation (%) (Median, IQR)**	91.5(89.5-94.3)	98(97-98)	<0.0001
**Treatment with lopinavir/ritonavir (n, %)**	30(88.2%)	109(87.2%)	0.798
**Treatment with lopinavir/ritonavir and**			
**hydroxychloroquine (n, %)**	1(2.9%)	15(12%)	0.07
**Body mass index (kg/m^2^) (Median, IQR)**	25.8 (23.4-27.6)	24.2 (22.1-26.1)	0.022
**Comorbidities**	**SC**	**MM**	**P value**
**Chronic hepatitis B virus (HBV)**	9	3	<0.0001
**Chronic HBV receiving entecavir**	1	2	0.517
**HBV-related cirrhosis**	0	0	a/n
**HBV cirrhosis**	0	0	a/n
**Hypertension**	13	11	<0.0001
**Diabetes**	4	10	0.492
**Coronary heart disease**	3	0	0.009
**Fatty liver**	1	1	0.321
**Other**	9	1	<0.0001

**Table 2:** Comparison of baseline demographics and clinical characteristics between SC and MM groups in the subset with liver function test abnormality.

**Characteristic**	**SC**	**MM**	**P value**
**Number (n, %)**	11 (32.4%)	17 (11.6%)	0.011
**Age (years) (Median, IQR)**	52 (40-63)	39 (30-47.0)	0.021
**Male gender (n, %)**	9 (81.8%)	11 (64.7%)	0.328
**Body mass index (kg/m^2^) (Median, IQR)**	26.2 (25.7-27.0)	24.5 (22.9-26.1)	0.120
**Comorbidities**	**SC**	**MM**	**P value**
**Chronic hepatitis B virus**	2 (1 on ETV)	1 (1 on ETV)	0.543
**Hepatitis B virus related cirrhosis**	0	0	a/n
**Hypertension**	3	1	0.269
**Diabetes**	1	1	1
**Other**	2	0	0.146
**The number of comorbidities**	**SC**	**MM**	**P value**
**One**	1	3	1
**Two**	3	0	0.05
**Three**	1	0	0.393

Values are expressed as median (interquartile range (IQR), 25-75%). P value is the comparison between severe/critical (SC) and mild/moderate (MM) patients. *P<0.05. ETV = entecavir.

### Liver function test abnormality frequency

Twenty-eight of the 159 (17.6%) hospitalized patients had LFT abnormality at the time of hospital admission (n=19), and a further 9 patients (5.7%) developed LFT abnormality during the first week of admission. The proportion of patients with LFT abnormality was significantly higher in the SC group compared to the MM group (32.4% vs 11.6%, p=0.011, Table 1).

**Figure 1:**
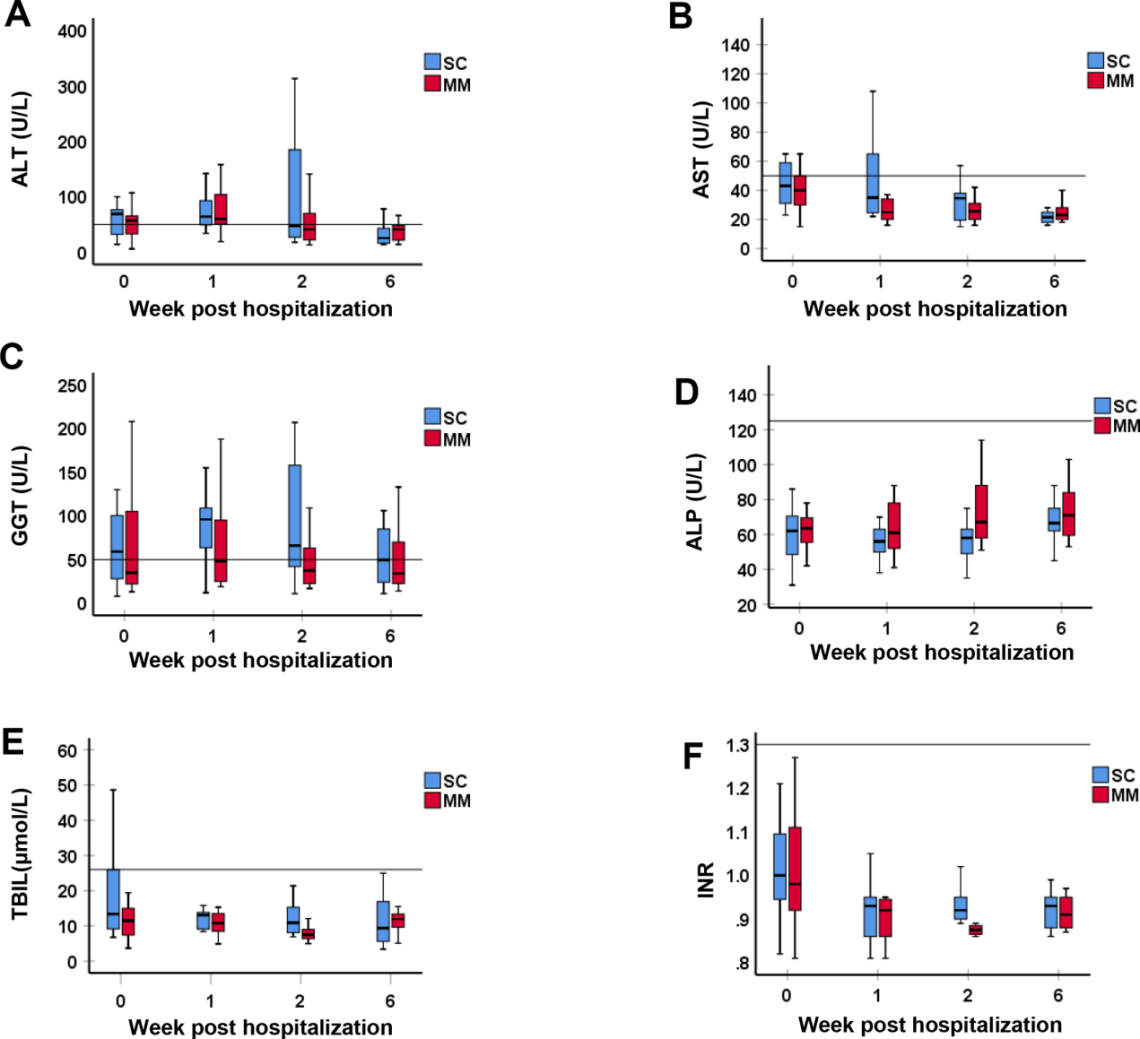
**Comparison of liver function test between MM and SC patient groups with liver function test abnormality.** The liver function tests including (**A**) ALT, (**B**) AST, (**C**) GGT, (**D**) ALP, (**E**) TBIL, and (**F**) INR, were compared between MM and SC patient groups with liver function test abnormality at Week 0, 1, 2 and 6 post hospitalization for COVID-19. Values are expressed as median (interquartile range (IQR), 25-75%). The horizontal line in each panel is the upper limit of normal (ULN) for each parameter. There was no statistically significant difference in any of the LFT or INR parameters between SC and MM patients.

Among patients with LFT abnormality, the median age was significantly higher in the SC group compared to the MM group (52 vs 39 years, p=0.021). Three patients had a history of chronic HBV infection, 2 of whom were receiving antiviral therapy with ETV (Tables 2, 3). The distribution of comorbidities was similar among the subset of patients with liver function test abnormality in the MM and SC groups, and in particular there was a similar number of patients with chronic HBV and patients receiving ETV in each group (Tables 2, 3 and Figure 1).

**Table 3:** Comparison of liver function test parameters between MM and SC group patients with abnormal liver function tests (Table 3-1) and normal liver function tests (Table 3-2).

**Table 3-1:** Patients with abnormal liver function within 1 week of admission.

		**Week 0**			**Week 1**			**Week 2**			**Week 6**		
	**NRR**	**SC n=11**	**MM n=17**	**P value**	**SC n=11**	**MM n=17**	**P value**	**SC n=11**	**MM n=17**	**P value**	**SC n=11**	**MM n=17**	**P value**
**ALT (U/L)**	0-50	69 (27-81)	62 (33.0-90.5)	0.962	70 (49-119)	60 (49-106)	0.64	47 (25-210)	41.0 (22-71.5)	0.378	25.5 (15.5-45.5)	42.5 (20.3-49.5)	0.291
**AST (U/L)**	0-50	46 (30-65)	40 (28-52.5)	0.423	37.50 (25.25-74.0)	24.0 (18.0-44.25)	0.078	34.50 (24.25-38.5)	25.0 (21.0-31.5)	0.128	21.5 (17.8-25.8)	23.5 (20-29)	0.178
**GGT (U/L)**	10-60	59 (21-130)	35 (21-108.5)	0.48	96 (55-114)	48 (24-99)	0.082	66 (41.5-166.5)	40 (22.5-79)	0.217	49.5 (23-87.5)	34 (22.3-73.5)	0.752
**ALP (U/L)**	45-125	62 (48-75)	64 (55-72)	0.495	56 (48-63)	61 (51-79)	0.232	58 (49-64)	67 (47-92.4)	0.045	66.5 (57.8-78.3)	71 (58.8-87)	0.598
**TBIL (μmmol/L)**	0-26	13.40 (8.6-33.1)	11.5 (7.2-15.5)	0.279	13.4 (8.6-33.1)	11.8 (8.5-15.3)	0.264	10.9 (7.8-18.4)	7.6 (6.3-12.6)	0.115	9.4 (5.6-16.9)	12 (9.5-13.5)	0.562
**INR**	0.94-1.30	1 (0.94-1.11)	0.98 (0.91-1.12)	0.925	0.93 (0.85-0.98)	0.92 (0.85-0.95)	0.744	0.92 (0.9-0.99)	0.88 (0.87-0.93)	0.176	0.93 (0.88-0.97)	0.91 (0.88-0.95)	0.735

Normal reference range (NRR), alanine aminotransferase (ALT), aspartate aminotransferase (AST), gamma-glutamyl transferase (GGT), alkaline phosphatase (ALP), total bilirubin (TBIL), and international normalized ratio (INR).

**Table 3-2:** Patients with normal liver function within 1 week of admission.

		**Week 0**			**Week 1**			**Week 2**			**Week 6**		
	**NRR**	**SC n=23**	**MM n=108**	**P**	**SC n=23**	**MM n=108**	**P**	**SC n=23**	**MM n=108**	**P**	**SC n=23**	**MM n=108**	**P**
**ALT (U/L)**	0-50	25 (18-33)	23 (13-36)	0.515	22 (18-32)	19 (13-34.5)	0.632	30 (22-47.5)	28.5 (15-44)	0.206	18 (13.5-33.8)	26 (15-40)	0.25
**AST (U/L)**	0-50	28 (23-30)	25 (19-31)	0.091	24 (19-26)	21 (18-24)	0.197	21 (18-28.5)	20 (16-26)	0.394	19 (16-26)	23 (18-30)	0.07
**GGT (U/L)**	10-60	23 (16-33)	26 (15-41)	0.934	23 (18-29)	22 (15-38)	0.55	30 (21-46.5)	26.5 (16.8-53)	0.416	21 (15.5-26.8)	29 (17-46)	0.099
**ALP (U/L)**	45-125	61 (46-66)	63 (51-73)	0.17	52 (45-58)	59 (48.5-70)	0.006	54 (40-65)	59 (51-72.3)	0.064	61 (51.8-74)	62 (53-77)	0.602
**TBIL (μmmol/L)**	0-26	11.4 (7.2-15.2)	9.9 (7.0-15.3)	0.495	13.6 (8.1-17.7)	11.9 (9.4-15.6)	0.951	9.8 (6.4-19.3)	7.5 (5.9-10.2)	0.034	11 (6.5-13)	10.5 (8.4-14.1)	0.562
**INR**	0.94-1.30	1.01 (0.95-1.07)	0.98 (0.94-1.07)	0.702	0.95 (0.93-1.01)	0.93 (0.88-1.0)	0.123	0.95 (0.88-1.08)	0.93 (0.9-0.95)	0.505	0.91 (0.87-0.97)	0.91 (0.89-0.92)	0.91

Values are expressed as median (interquartile range (IQR), 25-75%). P value is the comparison between Severe, Critical (SC) and Mild, Moderate (MM) group patients.

### Pattern and degree of liver function test abnormality

**Figure 2:**
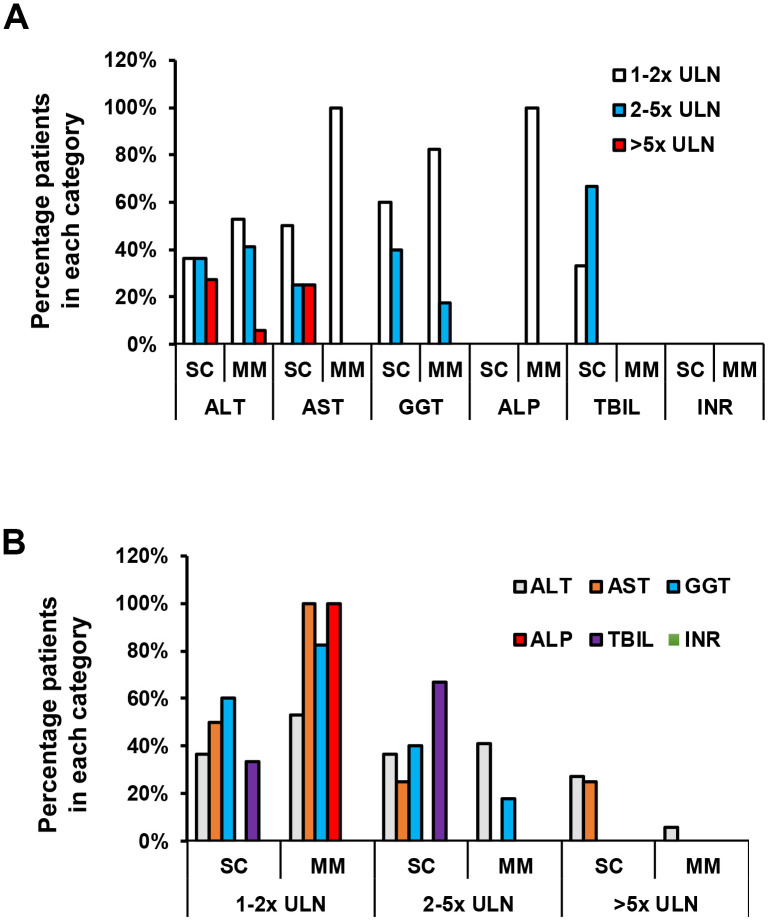
**Degree of liver function test abnormality in SC and MM groups in the subset with liver function test abnormality.** (**A**) Comparison of liver function abnormality between SC and MM groups with liver abnormality. (**B**) Comparison of liver function subset between SC and MM groups with liver abnormality. There was no statistically significant difference in the degree of LFT abnormality between SC and MM patients.

We analyzed the components of the LFT panel, including alanine aminotransferase (ALT) and aspartate aminotransferase (AST), which are markers of hepatocellular damage, and gamma-glutamyl transferase (GGT), and alkaline phosphatase (ALP), which are markers of cholestasis, in COVID-19 patients at the time admission (week 0), at 1, 2 and 6 weeks following date of admission as an inpatient or outpatient depending on length of admission. In addition, we analyzed markers of liver synthetic function including total bilirubin (TBIL), and coagulation profiles using the international normalized ratio (INR). We found that there was only a mild to moderate derangement in LFTs in both MM and SC patient groups (Tables 3, 4 and Figures 1, 2), with a mixed pattern of both hepatocellular injury and cholestasis (GGT elevations observed but no changes in ALP were observed), without significant liver synthetic dysfunction.

**Table 4:** Degree of liver function test abnormality in SC and MM groups in the subset with liver abnormality.

	**1-2 ULN**		**P value**	**2-5 ULN**		**P value**	**> 5ULN**		**P value**
	**SC**	**MM**		**SC**	**MM**		**SC**	**MM**	
**ALT (n, %)**	4(11,36.4%)	9(17,52.9%)	0.057	4(11,36.4%)	7(17, 41.2%)	0.799	3(11,27.3%)	1(17,5.9%)	0.269
**AST (n, %)**	4(8,50%)	7(7,100%)	0.799	2(8,25%)	0	0.543	2(8,25%)	0	0.146
**GGT (n, %)**	6(10,60%)	14(17,82.4%)	0.112	4(10,40%)	3(17,17.6%)	0.264	0	0	a/n
**ALP (n, %)**	0	1(100%)	0.206	0	0	a/n	**0**	**0**	a/n
**TBIL (n, %)**	1(3,33.3%)	0	0.206	2(3,66.7%)	0	0.068	0	0	a/n
**INR**	0	0	a/n	0	0	a/n	0	0	a/n

Alanine aminotransferase (ALT), aspartate aminotransferase (AST), gamma-glutamyl transferase (GGT), alkaline phosphatase (ALP), total bilirubin (TBIL), and international normalized ratio (INR).

Values are expressed as median (interquartile range (IQR), 25-75%). P value is the comparison between severe/critical (SC) and mild/moderate (MM) patients.

In more detail, the majority of patients had ALT, AST and GGT levels below 5 times the ULN (Table 4 and Figure 2). In the SC group, 4 (36.4%) patients had an ALT 1-2x ULN, 4 (36.4%) patients had an ALT 2-5x ULN, and 3 (27.3%) patients had an elevated ALT >5x ULN. In the MM group, 9 (52.9%) patients had an ALT 1-2x ULN, 7 (41.2%) patients had an ALT 2-5x ULN, and 1 (5.9%) patient had an ALT >5x ULN. AST was abnormal in 8 SC patients and 7 MM patients with LFT abnormalities. In the SC group, 4 (50%) patients had an AST of 1-2x ULN, 2 (25%) patients had AST 2-5x ULN, and 2 (25%) patients had an AST greater than 5 ULN (Table 4 and Figure 2). A total of 7 (100%) patients had an AST 1-2x ULN in the MM group, and no elevations >2x ULN were noted in this group (Table 4 and Figure 2). Median ALT and AST values failed to reach statistical significance between the SC and MM groups (Table 4). GGT levels were abnormal in 10 SC patients and 17 MM patients with LFT abnormalities (Table 4). In the SC group, 6 (60%) patients had elevated GGT levels 1-2x ULN, and 4 (40%) patients had elevated GGT levels 2-5x ULN. In the MM group, 14 (82.4%) patients had elevated GGT levels 1-2x ULN, and 3 (17.6%) patients had elevated GGT levels 2-5x ULN (Table 4 and Figure 2). Only 1 patient had an abnormal ALP in MM group (1-2x ULN), and no patients had abnormal ALP levels in the SC group (Table 4 and Figure 2). Only 3 patients had an elevated TBIL in the SC group; 1 patient had a TBIL 1-2x ULN, and 2 patients had a TBIL 2-5x ULN (all 3 patients had an elevated ALT, but normal ALP and INR) (Table 4 and Figure 2). TBIL elevation was not observed in the MM group. All patients had a normal INR (Tables 3, 4 and Figures 1, 2). Patients who experienced elevations in ALT above 2x ULN received glycyrrhizin therapy, which is routinely used as a hepatoprotective agent in our institution. LFT abnormalities recovered in all patients, and median time to normalization was 10 days.

In our case series, the most significantly elevated ALT was observed in a patient with chronic HBV who was treatment-naïve, where the peak ALT was 414 U/L, with an AST of 309 U/L, GGT of 290 U/L, ALP of 86 U/L, and an elevated TBIL of 70.5 μmol/L, but normal INR (1.08). Further characterization of the HBV revealed the patient was HBsAg positive, HBeAg positive and the HBV DNA was elevated at 22,800 IU/mL. Given the significant elevation in HBV DNA, the treating clinician felt that the LFT abnormalities were more likely attributable to their chronic HBV rather than COVID-19, and entecavir was commenced, in addition to glycyrrhizin therapy for 13 days. The LFTs normalized over the following 13 days in this patient.

## DISCUSSION

COVID-19 can lead to symptoms including fever, cough, fatigue, shortness of breath and myalgias. In more severe disease, COVID-19 may cause significant shortness of breath, hypoxia and respiratory failure, as well as radiographic features of pneumonia and/or other lung infiltrates. Although the lung is the primary target organ of SARS-CoV-2, confirmed at autopsy and characterized by an inflammatory reaction in the deep airway and alveolar injury [24], there are several reports that COVID-19 may also cause liver function test abnormality [1, 16, 17, 25], however these case series are difficult to interpret due to frequent co-administration of hepatotoxic agents such as lopinavir/ritonavir and other conditions that may lead to liver injury such as ischaemic hepatitis from severe and/or prolonged hypotension/shock. In this report, we observed that LFT abnormality is frequent in COVID-19 patients hospitalized at the No. 2 People’s Hospital of Fuyang City, with an overall frequency of LFT abnormality of 17.6% in the 159 patients with confirmed COVID-19. In addition, our study demonstrates that older patients are more likely to develop more severe COVID-19, which has been observed throughout the world, and are also more likely to develop LFT abnormality [18]. Furthermore, we observed a significantly higher proportion of patients with liver function test abnormality in the SC group compared to the MM group, suggesting a greater frequency of liver dysfunction in the SC group. These findings are consistent with other reports of COVID-19 in China [15, 25], and importantly demonstrates frequent LFT abnormality prior to the administration of potentially hepatotoxic agents. Our study therefore offers some interesting findings of liver involvement during COVID-19 infection.

Liver dysfunction has been seen during other respiratory virus pandemics, although the incidence of LFT dysfunction was more severe with pandemic A/H1N1 influenza in 2009 than during this current COVID-19 outbreak [26], whereby serum levels of AST, ALT, and GGT were significantly higher in the A/H1N1 influenza than observed in COVID-19. Interestingly, abnormalities in serum liver enzymes were strongly correlated with hypoxemia in the A/H1N1 influenza pandemic, suggesting that influenza itself may in some way mediate the hepatotoxicity [26]. When comparing liver function test parameters between our SC and MM COVID-19 patients, we found that the SC patients had a higher incidence of liver injury to MM patients, however the pattern and degree of LFT abnormality was not significantly different between the two groups with regards to the proportion of patients with LFT abnormalities 1-2x ULN, 2-5x ULN and >5x ULN. We did observe that median ALT and AST values were numerically higher in SC patients compared to MM patients, with median ALT values above the ULN in the SC group, however this failed to reach statistical significance. These findings indicate that COVID-19 is associated with mild to moderate liver function test abnormalities with a mixed picture of liver injury, particularly in SC patients. However, accompanying significant liver synthetic function compromise or liver failure were not observed in this cohort. In addition, our findings indicate that older patients are not only more likely to develop more severe COVID-19 but are also at greater risk of liver function abnormality.

It should be noted that the vast majority of the patients enrolled in this study received lopinavir/ritonavir with or without hydroxychloroquine as potential antiviral agents. Lopinavir/ritonavir is a well described to cause drug-induced liver injury (DILI). Therefore, we designed our study to restrict the definition of LFT abnormality to the first week following admission in order to limit potential confounding from hepatotoxicity from lopinavir/ritonavir. However, liver function parameters did not significantly change from baseline or week 1 to week 2, indicating that lopinavir/ritonavir-induced DILI does not explain our findings.

There are increasing reports of COVID-19 induced liver dysfunction in China, where mild elevations in liver functions tests have also been described [1, 15, 27, 28]. However, the mechanism by which COVID-19 induces liver function abnormality is not well characterized. There is much speculation regarding potential mechanisms, which include direct liver injury from SARS-CoV-2 infection of hepatocytes, cytokine storm syndrome, DILI and ischaemic hepatitis. We speculate that hepatocytes could be infected given SARS caused by SARS-CoV-1, another coronavirus similar to SARS-CoV-2, as SARS-CoV-1 RNA was detected in liver tissue from patients with SARS, although viral inclusions were not seen on electron microscopy [29].

Interestingly, non-specific histological features of microvascular steatosis and mild lobular and portal activity has been observed in the liver at autopsy in a patient who died of severe COVID-19 [27]. However, viral inclusions were not identified in liver tissue at autopsy and therefore it is unclear if these changes were related to direct viral infection of the liver by SARS-CoV-2, DILI, or even due to pre-existing fatty liver disease, although it should be noted that viral inclusions were also not seen in lung tissue (the primary target organ of COVID-19) in this patient. Another potential mechanism that has been considered is the effect of COVID-19 induced cytokine storm syndrome (CSS) on liver injury, but without strong evidence supporting this hypothesis. Liver damage may also be influenced by underlying liver diseases, such as chronic HBV and fatty liver disease, or as a result of pneumonia-associated hypoxia or ischaemic hepatitis from prolonged hypotension. These data highlight that further studies are required to elucidate the mechanism(s) of liver impairment in COVID-19.

In summary, we found that COVID-19 associated liver function test abnormality is more common in patients with severe or critical presentations of COVID-19, as well as older patients. Although the degree of COVID-19 induced liver function abnormality is relatively mild to moderate in our cohort without evidence of significant liver synthetic dysfunction or liver failure, it highlights the frequent incidence of LFT abnormalities in patients with COVID-19, which has implications for the management of these patients in order to preserve liver function with consideration of co-administration of hepatoprotective agents and to minimize exposure to hepatotoxic events, particularly in patients with underlying liver disease and older age. Our study adds to the growing body of evidence that SARS-CoV-2 is associated with liver function test abnormality, and particularly in older patients [18, 30, 31]. A more detailed understanding of the underlying mechanisms of liver injury from SAR-CoV-2, as well as viral pathogenesis and antiviral responses to COVID-19 are therefore required in order to best optimize older age patient outcomes.

## MATERIALS AND METHODS

### Patients and study design

As of March 4^th^, 2020, the No. 2 People’s Hospital of Fuyang City has admitted 159 patients (including 4 patients transferred from Bozhou City, Anhui Province) with confirmed COVID-19 since the outbreak of the disease in Anhui Province in January 2020. No COVID-19 related deaths have been recorded in this hospital. The majority of the patients enrolled in this study received lopinavir/ritonavir with or without hydroxychloroquine for antiviral therapy. All COVID-19 patients were diagnosed, classified and treated according to the guidelines of the Pneumonia Treatment Plan for the Novel Coronavirus Infection, National Health and Health Commission of the people’s Republic of China (Version 1-6) [19–22]. Patients with confirmed COVID-19 were included, and classification of severity criteria was as follows: 1) mild: mild clinical symptoms without pneumonia on imaging; 2) moderate: fever, respiratory tract infection symptoms with pneumonia on imaging; 3) severe: confirmed COVID-19 with one or more of the following 3 features: (a) breathing distress, respiratory rate ≥ 30 breaths/minute, (b) oxygen saturation ≤ 93% on room air, or (c) oxygenation index ≤ 300mmHg; 4) critical: confirmed COVID-19 with one or more of the following 3 features: (a) respiratory failure requiring mechanical ventilation, (b) coma, (c) combined organ failure requiring ICU monitoring (for example, dysfunction/failure of more than 2 organ systems that requires ICU support). Exclusion criteria included patients with respiratory symptoms that repeatedly tested negative for COVID-19 and did not have pneumonia on imaging, and those with new onset of liver dysfunction one week after hospitalization. This time point was chosen in order to exclude patients that may have developed abnormal LFTs from another cause such as ischaemic hepatitis from prolonged hypotension/shock or drug-induced liver injury. In this report, we sought to analyze the frequency of LFT abnormality and liver dysfunction in COVID-19 patients, and specifically to compare the incidence of LFT abnormality and liver dysfunction between COVID-19 patients with mild or moderate illness (MM group) and those with severe or critical illness (SC group). LFT abnormality is defined as any parameter of the liver enzyme panel greater than the upper limit of normal (ULN). We also evaluated liver synthetic function abnormality with International Normalized Ratio (INR) and elevated total bilirubin (TBIL).

### Ethics approval and consent to participate

This study was approved by the Ethics Review Committee of the No. 2 People’s Hospital of Fuyang City (reference number: 2020006) and was conducted in accordance with the ethical standards of the institutional and national research committees, and with the 1964 declaration of Helsinki. This study was registered at the Chinese Clinical Trial Registry (registration number ChiCTR2000031620).

### COVID-19 detection and laboratory parameter testing

Nasopharyngeal aspirates and sputum from patients with suspected COVID-19 were used for COVID-19 testing. COVID-19 RNA was detected by using real-time quantitative PCR (qPCR) (Shanghai BioGerm Medical Biotechnology Co., Shanghai, China). Liver function was measured by using the Hitachi 7600 fully automatic biochemical analyzer. The complete blood count was measured by using the SYSMEX CA5100 automatic clotting analyzer (Siemens Healthcare, Erlangen, Germany). Internationalized Normalized Ratio (INR) was calculated based on the prothrombin time (PT) test result.

### Statistical analyses

Data were analyzed using SPSS Statistics v25.0 (Armonk, New York, USA). Continuous data were expressed as medians with interquartile range, and categorical data as frequencies. Groups were compared using the Mann-Whitney U test, and the correlations between clinical, laboratory parameter were evaluated using the two-tailed chi-squared test.
